# Perioperative Inflammatory Cytokine Profiles in Patients with Atrial Fibrillation Undergoing Catheter Treatment: A Prospective Observational Study from Kazakhstan

**DOI:** 10.3390/jcm15135264

**Published:** 2026-07-06

**Authors:** Kenzhebek Bizhanov, Adil Baimbetov, Alexander Sapunov, Akmaral Izbassarova, Akmoldir Sarsenbayeva

**Affiliations:** 1 Department of Interventional Cardiology, Arrhythmology and Endovascular Surgery, Syzganov National Scientific Center of Surgery, Almaty 050004, Kazakhstan; kazpace@gmail.com (A.B.); alex.evasc@gmail.com (A.S.); akmoldir.sarsenbayeva@gmail.com (A.S.); 2 Faculty of Medicine and Healthcare, Al-Farabi Kazakh National University, Almaty 050040, Kazakhstan; 3 Department of Physical Medicine and Rehabilitation, Sports Medicine, Asfendiyarov Kazakh National Medical University, Almaty 050012, Kazakhstan; 4 Department of Cardiology, Asfendiyarov Kazakh National Medical University, Almaty 050012, Kazakhstan

**Keywords:** atrial fibrillation, interleukins, cytokines, catheter ablation, inflammation, Kazakhstan

## Abstract

**Background/Objectives**: Atrial fibrillation (AF) is the most common sustained cardiac arrhythmia worldwide, with inflammatory cytokines increasingly implicated in its development, persistence, and post-ablation recurrence. However, prospective perioperative cytokine data from Central Asia are lacking. The objective of the study is to evaluate temporal changes in inflammatory cytokines in AF patients undergoing catheter ablation and assess differences by AF type and recurrence status. **Methods**: In this prospective observational study conducted at the Syzganov National Scientific Center of Surgery, Almaty, Kazakhstan, 166 AF patients (mean age 66.1 ± 8.2 years; 57.8% male) were enrolled. Serum cytokines were measured using ELISA at baseline, immediately postablation, and at 6 months. Temporal changes were analysed using Friedman and Wilcoxon signed-rank tests, while between-group comparisons used Mann–Whitney U and Kruskal–Wallis tests. **Results**: IL-1β, IL-6, and IL-17A showed significant temporal changes (all *p* < 0.001). IL-6 increased immediately after ablation (0.088 to 0.122 pg/mL) before decreasing below baseline at 6 months (0.079 pg/mL). IL-17A progressively declined across all time points (0.073, 0.069, and 0.048 pg/mL). At 6 months, IL-1α levels were higher in primary versus recurrent AF (0.079 vs. 0.067 pg/mL; *p* = 0.047). Baseline IL-1β and IL-28A differed across AF subtypes (*p* = 0.040 and *p* = 0.014). No pre-operative cytokine independently predicted recurrence. **Conclusions**: AF ablation is associated with distinct cytokine trajectories, particularly for IL-1β, IL-6, and IL-17A. Cytokine profiles vary by AF type and recurrence, highlighting the potential role of immune monitoring in AF management. These findings should be regarded as exploratory and hypothesis-generating.

## 1. Introduction

Atrial fibrillation (AF) is the most common sustained cardiac arrhythmia, affecting an estimated 59 million individuals worldwide and representing 1–2% of the general population [1]. Its global burden is steadily increasing, driven by population ageing, the rising prevalence of cardiovascular risk factors, and improved detection rates. Across Asia, AF is anticipated to be present in at least 72 million individuals by 2050 [2]. The economic implications are substantial: annual treatment costs per patient have been estimated at $3624 on average and as high as $21,099 in high-income settings, generating national cost burdens of $6–26 billion in the United States [3,4]. Beyond direct costs, AF significantly increases the risk of ischemic stroke and transient ischemic attacks, with the population-attributable fraction of AF-related stroke ranging from 1.5% in adults aged 50–59 years to 23.5% in those aged 80–89 years [5]. These data underscore AF as a public health priority demanding improved mechanistic understanding and more effective therapeutic strategies.

Based on Global Burden of Disease 2019 data showing a rising global burden of atrial fibrillation, particularly in middle-income countries, Kazakhstan is likely to face a substantial AF burden, yet systematic epidemiological and immunological data remain scarce [6]. The National Scientific Centre of Surgery (NNCS) named after A.N. Syzganov is among the leading institutions in Kazakhstan delivering interventional arrhythmology services, including radiofrequency and cryoballoon catheter ablation. Despite accumulating procedural experience and promising clinical outcomes reported in single-center studies [7,8], several critical questions remain unanswered: which patients are at highest risk for AF recurrence after ablation, and what immunological mechanisms underlie treatment failure? Recurrent atrial arrhythmia occurs in 33% and 43% of ablated patients at 12 and 24 months, respectively, with no significant survival-free-from-arrhythmia difference between ablation strategies [9]. A gap persists in understanding the specific role of the perioperative cytokine milieu in determining these outcomes within Central Asian populations, who may exhibit distinct risk factor profiles, genetic backgrounds, and healthcare access patterns compared to Western cohorts.

Inflammatory pathways have emerged as pivotal in the pathogenesis of AF. Atrial inflammation, fibrosis, and electrical remodeling are inter-related processes mediated by a network of pro- and anti-inflammatory cytokines. Interleukin-6 (IL-6), one of the most studied cytokines in this context, significantly promotes atrial fibrosis through multiple signaling cascades and is elevated in AF patients compared to those in sinus rhythm [10]. Interleukin-1β (IL-1β) promotes rapid atrial electrical remodeling and is more strongly associated with persistent than paroxysmal AF, suggesting a dose–response relationship between inflammatory burden and AF chronicity [11]. IL-17A, a key pro-inflammatory cytokine released by T-helper 17 cells, facilitates recruitment of inflammatory cells to atrial tissue and has been implicated in the pathogenesis of atrial structural remodeling [12]. The IL-12/IL-23 axis modulates adaptive immunity and may contribute to the fibrotic substrate that perpetuates AF [13]. Despite these insights, data on how these cytokines change dynamically across the perioperative period—specifically before ablation, immediately after the procedure, and at six months follow-up—and whether such trajectories predict recurrence, remain limited, particularly in non-Western populations.

The present study therefore aims to characterize the perioperative temporal dynamics of serum IL-1α, IL-1β, IL-6, IL-17A, IL-12/IL-23, and IL-28A in a cohort of Kazakhstani AF patients undergoing catheter ablation; to compare cytokine profiles by AF type (paroxysmal, persistent, long-standing persistent, permanent) and episode status (primary versus recurrent AF); and to evaluate the predictive value of pre-operative cytokine levels for AF recurrence using multivariable regression analysis. The findings are intended to contribute to the development of immunological monitoring algorithms that can guide clinical decision-making in the interventional management of AF.

## 2. Materials and Methods

### 2.1. Study Design and Setting

This was a prospective, observational, single-center study conducted in the Department of Interventional Cardiology, Arrhythmology and Endovascular Surgery (RHiCA) at the Syzganov National Scientific Centre of Surgery (NNCS), Almaty, Republic of Kazakhstan. The study was conducted as part of a grant-funded research program approved by the Ministry of Science and Higher Education of Kazakhstan (project period: January 2024–December 2026). Data collection spanned the clinical activity of the department over the study recruitment period.

### 2.2. Participants and Sampling

Consecutive patients admitted to the RHiCA department for interventional treatment of AF (catheter ablation—radiofrequency or cryoballoon) were screened for eligibility using a continuous (census) sampling approach. The target sample size was calculated at a 95% confidence level with a ±5% margin of error, based on an estimated annual department caseload of 150 AF patients, yielding a required sample of 108. The final enrolled cohort comprised 166 patients, exceeding the minimum requirement. All participants provided written informed consent prior to enrolment.

### 2.3. Inclusion and Exclusion Criteria

Inclusion criteria: (1) confirmed diagnosis of AF (paroxysmal, persistent, long-standing persistent, or permanent) based on ECG documentation and international guidelines; (2) indication for interventional treatment (catheter ablation); (3) age ≥ 18 years; (4) written informed consent. Indications for catheter ablation followed contemporary guideline recommendations (2020 ESC and 2023 ACC/AHA/ACCP/HRS guidelines): symptomatic paroxysmal or persistent AF that was refractory or intolerant to at least one class I or III antiarrhythmic drug, or, in selected symptomatic patients, as first-line rhythm-control therapy after shared decision-making.

Exclusion criteria: (1) acute inflammatory or infectious conditions at time of enrolment; (2) systemic autoimmune disorders requiring active immunosuppressive therapy; (3) active malignancy; (4) severe renal or hepatic impairment (eGFR < 30 mL/min/1.73 m^2^ or Child-Pugh C); (5) refusal to provide informed consent; (6) inability to attend follow-up assessments.

### 2.4. Variable Definitions

The primary exposure variables were serum concentrations of six cytokines—IL-1α, IL-1β, IL-6, IL-17A, IL-12/IL-23, and IL-28A—measured in picograms per milliliter (pg/mL) at three time points: (1) at hospital admission, prior to the ablation procedure; (2) on the first postoperative day following ablation; and (3) at six-month outpatient follow-up. Clinical variables collected included: age (years), sex, body height (cm) and weight (kg), body mass index (BMI, calculated as weight/height^2^), AF episode status (primary or recurrent AF), AF type (paroxysmal, persistent, long-standing persistent, permanent), and comorbidities (type 2 diabetes mellitus, hepatitis B and C serostatus). Recurrent AF was defined as documented recurrence of atrial arrhythmia after a prior ablation procedure.

AF type was classified per the 2020 ESC Guidelines: paroxysmal AF (self-terminating, usually within 48 h and no later than 7 days); persistent AF (continuously sustained beyond 7 days, including episodes terminated by cardioversion after ≥7 days); long-standing persistent AF (continuous AF ≥ 12 months once a rhythm-control strategy is adopted); and permanent AF (AF accepted by patient and physician, with no further attempts to restore or maintain sinus rhythm). In this study, “primary AF” denoted patients undergoing their first (index) catheter ablation, whereas “recurrent AF” denoted documented recurrence of any atrial tachyarrhythmia (AF, atrial flutter or atrial tachycardia) lasting >30 s after the 3-month post-procedural blanking period in patients with a prior ablation; index (first-time) ablation is the standard initial interventional therapy for symptomatic AF at our centre, which explains the predominance of primary-AF cases. Acute procedural success was defined as confirmed electrical isolation of all pulmonary veins (entrance and, where assessed, exit block) at the end of the procedure. No patient was excluded post hoc on the basis of acute procedural success or subsequent arrhythmia response; all enrolled patients with available samples were retained, so that responders and non-responders are both represented in the analysed cohort.

### 2.5. Cytokine Measurement

Blood samples (serum) were collected at each of the three pre-defined time points by trained nursing staff. Serum was separated by centrifugation and stored at −80 °C until analysis. Quantitative enzyme-linked immunosorbent assay (ELISA) for IL-1α, IL-1β, IL-6, IL-17A, IL-12/IL-23, and IL-28A was performed using a Bio-Rad ELISA analyzer complex (Bio-Rad Laboratories, Marnes-la-Coquette, France, 2014) at the clinical laboratory of NNCS, in accordance with the manufacturer’s protocols. Results were expressed in pg/mL.

### 2.6. Statistical Analysis

Statistical analyses were performed using SAS OnDemand for Academics (version 3.81, Cary, NC, USA). Continuous variables were assessed for normality using visual inspection of histograms and Q-Q plots. Given the non-normal distribution of most cytokine measurements, non-parametric methods were used throughout. Continuous variables are presented as median [interquartile range, IQR], and categorical variables as absolute frequencies and percentages.

To assess temporal within-subject changes in cytokine levels across the three time points, the Friedman test was applied in the subset of patients with complete data at all three assessments (n = 110). Post-hoc pairwise comparisons were performed using the Wilcoxon signed-rank test (Bonferroni correction for multiple comparisons was considered; uncorrected *p*-values are reported with significance threshold set at *p* < 0.05). Group comparisons between primary and recurrent AF were performed using the Mann–Whitney U test for continuous variables and chi-squared test for categorical variables. Differences across AF types (paroxysmal, persistent, long-standing persistent, permanent) were tested using the Kruskal–Wallis test.

Multivariable binary logistic regression was used to identify independent predictors of recurrent AF (versus primary AF). Cytokine values were natural log-transformed [log (1 + x)] prior to regression to approximate normality. Adjusted odds ratios (aORs) with 95% confidence intervals (CIs) are reported. Model fit was assessed using the log-likelihood ratio test and the McFadden pseudo-R^2^. A two-tailed *p*-value < 0.05 was considered statistically significant throughout.

Handling of missing data and analysis populations. Missing values were not imputed; each analysis used all available data for the variables involved. The within-subject temporal analysis (Friedman and Wilcoxon tests) was restricted to the 110 patients with complete cytokine measurements at all three time points, whereas the cross-sectional comparisons by AF type and by recurrence status used every patient with a non-missing value at the relevant time point; denominators therefore differ between analyses and are reported for each comparison. Patients with missing AF type (n = 37) or missing AF episode status (n = 3) were excluded only from the specific stratified analyses that required those variables.

Multiplicity. For the within-subject post-hoc pairwise tests a Bonferroni-adjusted threshold was applied (three comparisons per cytokine; adjusted α = 0.017), and for the between-group comparisons across the six cytokines a Benjamini–Hochberg false-discovery-rate (FDR) correction was applied. Associations that did not survive correction—including the 6-month IL-1α difference between primary and recurrent AF (uncorrected *p* = 0.047; FDR-adjusted *p* = 0.28) and the IL-1β admission-versus-follow-up and IL-17A admission-versus-post-operative pairwise comparisons (both uncorrected *p* = 0.019, above the Bonferroni threshold)—are identified as such and interpreted with caution.

During the preparation of this work, the authors used ChatGPT (OpenAI, GPT-5.5) to assist reference formatting. The authors have reviewed and edited the output and take full responsibility for the content of this publication.

### 2.7. Ethics

The study protocol was approved by the Local Ethics Committee (LEC) of the Syzganov NNCS (Protocol No. 4, dated 10 November 2023). All procedures were conducted in accordance with the principles of the Declaration of Helsinki. All participants provided written informed consent. Patient data were anonymized and stored securely in accordance with applicable data protection regulations.

## 3. Results

### 3.1. Patient Characteristics

A total of 166 patients with AF were included. The cohort comprised 96 males (57.8%) and 70 females (42.2%), with a median age of 67 years [IQR: 62–72] and median BMI of 29.3 kg/m^2^ [IQR: 25.3–33.9], consistent with overweight status. Of the cohort, 127 patients (76.5%) had primary AF and 36 (21.7%) had recurrent AF. The most prevalent AF types were persistent (29.5%) and paroxysmal AF (26.5%), followed by long-standing persistent (15.7%) and permanent (6.0%); AF type was not recorded for 37 patients (22.3%). Comorbidities included diabetes mellitus in 21 patients (12.7%), hepatitis B in 4 (2.4%), and hepatitis C in 3 (1.8%). No statistically significant differences in age, sex, BMI, or diabetes prevalence were observed between primary and recurrent AF groups (all *p* > 0.14; Mann–Whitney U and chi-squared tests). Full baseline characteristics are summarized in Table 1. AF episode status (primary vs. recurrent) was available for 163 patients (127 primary, 36 recurrent) and was not documented for 3 patients (1.8%); AF type was additionally not documented for 37 patients (22.3%). These patients were retained in all analyses for which their data were available and were omitted only from the specific stratified comparisons requiring the missing variable. The mean age of 66.1 ± 8.2 years (median 67) is consistent with reported AF catheter-ablation cohorts, in which the ablated population is typically younger than the overall AF population because older patients more often have permanent AF or comorbidities favoring a rate-control strategy.

### 3.2. Temporal Dynamics of Cytokines Across the Perioperative Period

Friedman tests in the 110 patients with complete cytokine data at all three time points revealed significant perioperative temporal changes for IL-1β (χ^2^ = 55.51, *p* < 0.001), IL-6 (χ^2^ = 53.23, *p* < 0.001), and IL-17A (χ^2^ = 25.28, *p* < 0.001). No significant temporal variation was observed for IL-1α, IL-12/IL-23, or IL-28A (all *p* > 0.14).

**Figure 1:**
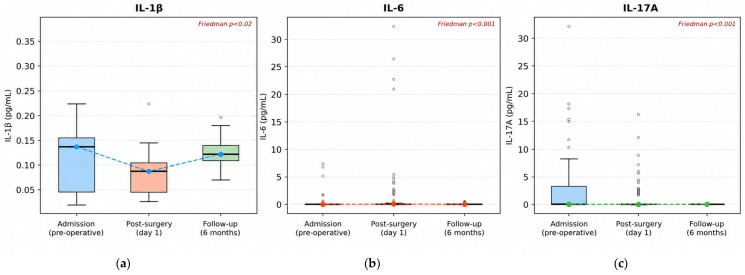
Temporal changes in IL-1β (**a**), IL-6 (**b**), and IL-17A (**c**) across the perioperative period (pre-operative admission, post-operative day 1, and 6-month follow-up) in 110 patients with complete data at all three time points. Boxes represent IQR; horizontal lines indicate medians; dashed lines connect median values across time points.

Post hoc pairwise Wilcoxon signed-rank tests demonstrated that IL-1β decreased significantly from admission to the post-operative day (median from 0.137 to 0.087 pg/mL; *p* < 0.001) and then partially recovered by six-month follow-up (from 0.087 to 0.121 pg/mL; *p* < 0.001); the comparison between admission and follow-up also remained statistically significant (*p* = 0.019), indicating that IL-1β at six months remained modestly but significantly lower than at baseline. IL-6 increased markedly from admission to the early post-operative period (from 0.088 to 0.123 pg/mL; *p* < 0.001), consistent with an acute inflammatory response to the ablation procedure, before declining significantly below pre-operative levels at six-month follow-up (from 0.123 to 0.079 pg/mL; *p* < 0.001; admission vs. follow-up: *p* = 0.010). IL-17A demonstrated a sustained and progressive decline across all three time points (admission: 0.073; post-surgery: 0.069; follow-up: 0.048 pg/mL; all pairwise comparisons *p* ≤ 0.019), suggesting a progressive dampening of the Th17-mediated inflammatory response following successful ablation. These temporal patterns are displayed graphically in Figure 1 and summarized in Table 2. Here and throughout, “following ablation” refers to the post-procedural course of the whole enrolled cohort and does not imply selection of acutely successful cases; no patient was excluded on the basis of procedural success or arrhythmia recurrence.

### 3.3. Cytokine Profiles by AF Type

**Figure 2:**
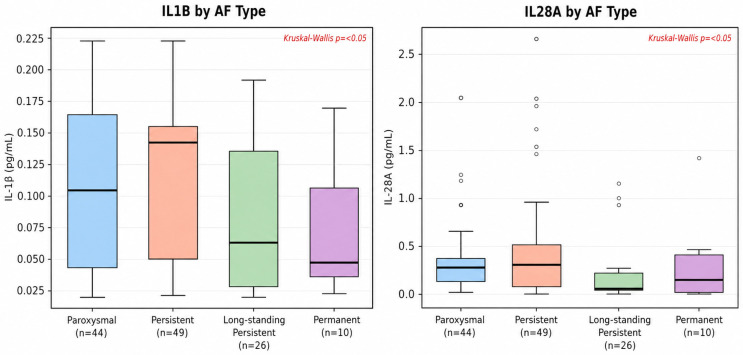
IL-1β (**left**) and IL-28A (**right**) at hospital admission stratified by atrial fibrillation type. Boxes represent IQR; lines indicate medians. Kruskal–Wallis: IL-1β *p* = 0.040; IL-28A *p* = 0.014.

Kruskal–Wallis tests revealed significant differences in cytokine levels across AF types at the time of admission. IL-1β differed significantly across paroxysmal, persistent, long-standing persistent, and permanent AF (H = 8.29, *p* = 0.040). Post hoc inspection indicated that persistent AF was associated with the highest median IL-1β (0.142 pg/mL), followed by paroxysmal (0.104 pg/mL), long-standing persistent (0.064 pg/mL), and permanent (0.047 pg/mL) forms, suggesting a non-linear relationship between AF chronicity and IL-1β. IL-28A at admission also differed significantly across AF types (H = 10.58, *p* = 0.014), with the highest levels in persistent AF (0.323 pg/mL) and paroxysmal AF (0.287 pg/mL), and markedly lower levels in long-standing persistent (0.071 pg/mL) and permanent AF (0.164 pg/mL). In the post-operative period, significant variation by AF type was observed for IL-1α (H = 11.32, *p* = 0.010), which was notably higher in long-standing persistent (11.010 pg/mL) and permanent AF (3.110 pg/mL) compared to paroxysmal (0.077 pg/mL) and persistent (0.061 pg/mL) forms. IL-1β post-operatively also differed significantly by AF type (H = 8.09, *p* = 0.044). At six-month follow-up, IL-1α continued to show significant variation by AF type (H = 8.61, *p* = 0.035), being highest in paroxysmal (0.082 pg/mL) and lowest in permanent AF (0.058 pg/mL). These findings are illustrated in Figure 2. The post-operative IL-1α medians in the long-standing persistent (11.010 pg/mL) and permanent (3.110 pg/mL) groups are correct as reported but reflect a markedly bimodal post-procedural distribution: within each AF-type group a subset of patients showed very high values (up to ~88 pg/mL) while others remained near the lower limit (~0.05 pg/mL), producing wide interquartile ranges (long-standing persistent IQR 0.068–26.380; permanent IQR 0.068–26.170). This dispersion should be borne in mind when interpreting the medians, and the comparison is reported as exploratory.

### 3.4. Cytokine Differences by AF Recurrence Status

**Figure 3:**
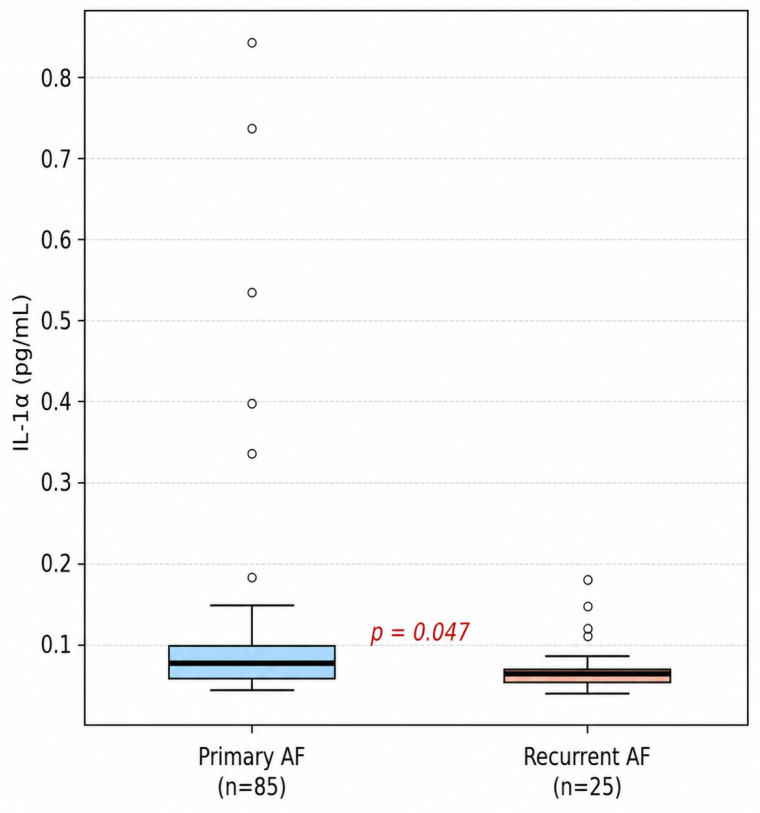
IL-1α concentrations at 6-month follow-up in patients with primary versus recurrent atrial fibrillation. Median values shown; boxes represent IQR. Mann–Whitney U test, *p* = 0.047.

Comparisons between primary (n = 127) and recurrent (n = 36) AF groups revealed no significant differences in cytokine levels at the time of hospital admission or in the early post-operative period (all *p* > 0.05). However, at six-month follow-up, IL-1α was significantly higher in patients with primary AF compared to those with recurrent AF (median 0.079 [IQR: 0.062–0.100] vs. 0.067 [IQR: 0.057–0.073] pg/mL; Mann–Whitney U = 1341.0, *p* = 0.047), as shown in Figure 3. This suggests that patients who experienced AF recurrence maintained lower IL-1α concentrations at the 6-month time point, potentially reflecting a distinct immunological trajectory following the ablation procedure. This 6-month IL-1α difference was of borderline significance and did not remain significant after Benjamini–Hochberg correction for the six cytokines tested (FDR-adjusted *p* = 0.28); it should therefore be regarded as hypothesis-generating rather than a robust association.

### 3.5. Multivariable Logistic Regression: Predictors of Recurrent AF

Multivariable logistic regression was performed to assess whether pre-operative cytokine levels (log-transformed) independently predicted AF recurrence after adjustment for age, sex, BMI, and diabetes mellitus. The overall model demonstrated limited predictive performance (McFadden pseudo-R^2^ = 0.049; LLR *p* = 0.509). None of the individual cytokines at admission reached statistical significance as independent predictors of recurrent AF after adjustment. Similarly, age, sex, BMI, and diabetes were not significantly associated with recurrence in this cohort. Detailed results of the multivariable model are presented in Table 3. Given the limited model performance (pseudo-R^2^ = 0.049; LLR *p* = 0.509) and the small recurrent subgroup (n = 36), these regression results are underpowered and do not support the use of a single baseline cytokine for clinical prediction of recurrence.

## 4. Discussion

### 4.1. Main Findings and Possible Explanations

This prospective observational study characterizes, for the first time from a Kazakhstani cohort, the perioperative inflammatory cytokine trajectory in patients with AF undergoing catheter ablation. The key findings can be summarized as follows: (1) IL-1β, IL-6, and IL-17A exhibit significant and biologically coherent temporal changes across the perioperative period; (2) IL-1β and IL-28A at admission differ significantly across AF types, with patterns suggesting a non-linear relationship between disease chronicity and baseline inflammatory burden; (3) IL-1α at six-month follow-up is significantly lower in patients with recurrent compared to primary AF, providing a potential immunological signal of treatment failure; and (4) no pre-operative cytokine independently predicts AF recurrence in multivariable analysis, suggesting that single-timepoint immunological profiling may be insufficient for clinical prediction. These observations are exploratory and hypothesis-generating; given the single-centre design, moderate sample size, incomplete longitudinal sampling and multiplicity of comparisons, they require confirmation in larger multicentre studies before any clinical application.

The biphasic pattern of IL-6 observed in the present study—an acute post-procedural rise followed by a significant reduction below pre-operative levels at six months—is consistent with the well-established role of IL-6 as an acute-phase cytokine and its longer-term downregulation following successful rhythm control. A large body of evidence links elevated IL-6 with AF perpetuation through multiple mechanisms, including atrial fibrosis, cardiomyocyte hypertrophy, and disruption of gap-junction proteins [10,14]. The post-ablation IL-6 rise likely reflects procedural tissue injury and an acute sterile inflammatory response, a phenomenon observed in published series of both radiofrequency and cryoablation [15]. The subsequent decline below pre-operative baseline at six months is reassuring and consistent with restoration of normal sinus rhythm reducing the chronic inflammatory state that sustains AF. The progressive and monotonic decline in IL-17A across all three time points observed in our cohort has not been comprehensively described in prior literature. IL-17A mediates recruitment of neutrophils and other inflammatory cells to the atrial wall, contributing to structural remodeling and fibrosis [12]. Its progressive reduction may signal attenuation of the Th17-driven inflammatory circuit following ablation, which could represent one mechanistic pathway through which catheter ablation exerts its long-term anti-arrhythmic effects.

The association of higher IL-1β levels at admission with persistent AF (versus lower levels in long-standing persistent and permanent forms) is particularly intriguing and may reflect an inverted-U relationship between AF duration and acute inflammatory activity. Early and mid-stage AF forms may sustain an actively inflamed atrial substrate, while advanced AF (long-standing persistent, permanent) may have transitioned to a predominantly fibrotic rather than inflammatory phenotype, thus exhibiting lower circulating IL-1β. This hypothesis aligns with the pathophysiological concept of ‘inflammageing’ of the atrium, in which sustained inflammatory activation eventually gives way to a ‘burnt-out’ fibrous remodeling [11]. IL-28A (IFN-λ2), whose role in AF pathogenesis has been characterized as largely unknown in the grant proposal literature review, demonstrated significant variation across AF types at admission, being highest in paroxysmal and persistent forms. Although the mechanistic basis for this finding requires further investigation, IL-28A belongs to the type III interferon family with antiviral and immunomodulatory properties; its elevation in earlier AF forms may reflect active immune surveillance or residual innate immune activation preceding structural remodeling.

Our temporal findings can be placed alongside prior reports. The acute post-procedural rise in IL-6 we observed mirrors data from radiofrequency and cryoballoon series in which IL-6 and CRP peak within 1–3 days of ablation and then decline, reflecting procedural tissue injury, and the colchicine trials of Deftereos et al., in which attenuating this inflammatory surge reduced early recurrence [15,16,17]. Baseline IL-6 elevation in AF versus sinus rhythm, and its association with recurrence, has been reported in several cohorts and meta-analyses, consistent with the mechanistic role we describe [18,19,20]. For IL-1β, experimental work linking NLRP3-inflammasome activation to atrial remodelling (Yao et al.) supports our observation of higher baseline IL-1β in earlier AF forms, although the non-linear pattern across AF chronicity we found has been less consistently described and warrants replication [12,21]. Data on IL-17A and IL-28A (IFN-λ2) in human AF remain sparse, so the progressive IL-17A decline and the AF-type differences in IL-28A reported here add novel, though preliminary, observations to a limited literature.

The lower IL-1α levels at six-month follow-up in patients with recurrent AF compared to those with primary AF raises important clinical hypotheses. Rather than a protective signal, this finding may indicate that recurrent AF patients—who have typically already undergone prior ablation(s)—enter a state of attenuated innate inflammatory responsiveness, possibly due to repeated procedural injury or a fundamentally different immunological set-point. Alternatively, primary AF patients retaining higher IL-1α at six months may represent an ongoing, yet controlled, adaptive immune response associated with successful healing and fibrosis resolution. This differential trajectory was not captured at admission or post-operatively, reinforcing the value of delayed immunological assessment in evaluating ablation outcomes. The absence of statistically significant pre-operative cytokine predictors of recurrence in multivariable regression likely reflects the limitations of sample size, the heterogeneity within the recurrent AF group, and the inherent complexity of immune regulation in AF. Single-cytokine, single-timepoint analyses are insufficient to capture the multi-dimensional immunological landscape required for accurate clinical prediction.

### 4.2. Implications of the Study Results

The findings of this study carry several forward-looking clinical and research implications. First, they argue for the incorporation of perioperative cytokine monitoring—specifically IL-6, IL-1β, and IL-17A—into structured follow-up protocols for AF patients undergoing ablation, as these markers provide mechanistic insights beyond standard hemodynamic or ECG-based assessments. Second, the differential cytokine trajectories across AF types suggest that personalized immunological profiling at the time of ablation may help stratify patients by inflammatory phenotype, potentially informing decisions about adjunctive anti-inflammatory therapies. Colchicine, IL-6 receptor antagonists, and IL-1 pathway inhibitors have shown preliminary efficacy in reducing post-ablation inflammatory responses and potentially improving long-term outcomes [22]; cytokine profiling could identify patients most likely to benefit from these approaches. Third, the establishment of Kazakhstan-specific immunological preliminary reference data for AF patients is foundational for future multicenter studies, national registries, and the development of local clinical guidelines aligned with international standards. These implications are aspirational and contingent on external validation; the present data do not yet justify changes to clinical management, and cytokine monitoring should at this stage be confined to the research setting.

### 4.3. Study Limitations

Several limitations of this study should be acknowledged. First, the single-center design limits generalizability of the findings to the broader Kazakhstani and Central Asian AF population. Second, a substantial proportion of patients lacked complete cytokine measurements at all three time points (n = 110 with complete data out of 166 enrolled), primarily due to loss to follow-up at six months, introducing potential selection bias in the temporal analyses. Third, the study did not systematically record the specific ablation modality (radiofrequency vs. cryoballoon) or procedural parameters (ablation time, lesion number, pulmonary vein isolation success), which could confound the cytokine trajectories observed post-procedure. Fourth, the recurrent AF group was relatively small (n = 36), limiting the statistical power of group comparisons and regression analyses. Fifth, residual confounding cannot be excluded given the observational design; factors such as concomitant medications (particularly anti-inflammatory, anticoagulant, and antiarrhythmic drugs), dietary factors, and background inflammatory conditions were not fully controlled. Finally, the absence of a non-ablated AF comparison group or a sinus rhythm control group limits the ability to attribute cytokine changes specifically to the procedure versus the natural history of AF. In addition, the analysis involved multiple cytokines, time points and subgroup comparisons; although Bonferroni and false-discovery-rate corrections were applied, some nominally significant associations (notably the borderline 6-month IL-1α difference) did not survive correction and may represent chance findings. The small recurrent-AF subgroup (n = 36) further limits the reliability of recurrence-based comparisons. Accordingly, all findings are best interpreted as exploratory and hypothesis-generating rather than as evidence for immediate clinical application.

## 5. Conclusions

In conclusion, this prospective observational study provides the first systematic characterization of perioperative cytokine dynamics in Kazakhstani AF patients undergoing catheter ablation. IL-1β, IL-6, and IL-17A exhibit statistically significant and biologically interpretable temporal changes that are consistent with the inflammatory pathophysiology of AF and the procedural injury–repair cycle of catheter ablation. IL-1β and IL-28A differ significantly across AF types, suggesting immunological heterogeneity across the disease spectrum. IL-1α at six-month follow-up is lower in patients with recurrent versus primary AF, providing a potentially informative immunological signal of treatment failure. These findings support the integration of cytokine monitoring into AF management frameworks and establish a foundation for larger multi-center prospective studies and the development of evidence-based immunological algorithms for the assessment of ablation efficacy in Kazakhstan and the Central Asian region. These conclusions are preliminary and hypothesis-generating; rather than supporting current clinical use of cytokine monitoring, they are intended to motivate and inform larger, adequately powered, multicentre studies that can formally test the predictive value of perioperative inflammatory profiling.

## Figures and Tables

**Table 1:** Baseline clinical and immunological characteristics of the study population.

Variable	Overall (N = 166)	Primary AF (n = 127)	Recurrent AF (n = 36)
Age, years—median [IQR] *	67 [62–72]	67 [62–72]	67 [61–71]
Male sex—n (%) *	96 (57.8%)	75 (59.1%)	18 (50.0%)
BMI, kg/m^2^—median [IQR] *	29.3 [25.3–33.9]	29.3 [24.9–33.9]	29.3 [25.7–33.1]
Diabetes mellitus—n (%) *	21 (12.7%)	19 (15.0%)	2 (5.6%)
Hepatitis B—n (%)	4 (2.4%)	NA	NA
Hepatitis C—n (%)	3 (1.8%)	NA	NA
AF type—n (%)			
Paroxysmal	44 (26.5%)	—	—
Persistent	49 (29.5%)	—	—
Long-standing persistent	26 (15.7%)	—	—
Permanent	10 (6.0%)	—	—
Cytokines at admission—median [IQR] (pg/mL)			
IL-1α	0.076 [0.053–22.15]	0.083 [0.052–22.00]	0.075 [0.054–21.89]
IL-1β	0.133 [0.043–0.155]	0.130 [0.042–0.154]	0.139 [0.048–0.161]
IL-6	0.088 [0.070–0.120]	0.087 [0.070–0.121]	0.092 [0.071–0.118]
IL-17A	0.074 [0.059–4.240]	0.077 [0.060–4.691]	0.065 [0.056–0.866]
IL-12/IL-23	273.5 [150.8–639.9]	276.0 [157.4–641.4]	222.9 [136.5–467.2]
IL-28A	0.275 [0.058–0.435]	0.274 [0.058–0.445]	0.290 [0.103–0.415]

Data are presented as median [IQR] for continuous variables and n (%) for categorical variables; *, all *p* > 0.14 (Mann–Whitney U and chi-squared tests); AF, atrial fibrillation; BMI, body mass index; IQR, interquartile range; IL, interleukin; NA, not applicable.

**Table 2:** Perioperative cytokine levels (median [IQR]) and results of Friedman test for temporal change (n = 110 complete cases).

Cytokine (pg/mL)	Admission (n = 165)	Post-Surgery (n = 145)	6-Month Follow-Up (n = 112)	Friedman *p*-Value
IL-1α	0.076 [0.053–22.15]	0.069 [0.055–12.81]	0.073 [0.059–0.098]	0.907
IL-1β	0.133 [0.043–0.155]	0.087 [0.046–0.103]	0.121 [0.109–0.140]	<0.001 †
IL-6	0.088 [0.070–0.120]	0.122 [0.096–0.177]	0.079 [0.064–0.100]	<0.001 †
IL-17A	0.074 [0.059–4.240]	0.071 [0.051–0.106]	0.048 [0.039–0.062]	<0.001 †
IL-12/IL-23	273.5 [150.8–639.9]	247.1 [156.0–409.1]	223.1 [162.0–269.9]	0.145
IL-28A	0.275 [0.058–0.435]	0.213 [0.083–0.389]	0.283 [0.216–0.567]	0.168

All values in pg/mL; IQR, interquartile range. †, *p* < 0.001 by Friedman test; Pairwise post-hoc: IL-1β: admission vs. post-surgery *p* < 0.001, post-surgery vs. follow-up *p* < 0.001, admission vs. follow-up *p* = 0.019; IL-6: admission vs. post-surgery *p* < 0.001, post-surgery vs. follow-up *p* < 0.001, admission vs. follow-up *p* = 0.010; IL-17A: admission vs. post-surgery *p* = 0.019, post-surgery vs. follow-up *p* = 0.001, admission vs. follow-up *p* < 0.001.

**Table 3:** Multivariable logistic regression analysis for recurrent AF (n = 164; pseudo-R^2^ = 0.049).

Variable	aOR	95% CI	*p*-Value
Age (per 1 year)	0.987	0.942–1.035	0.591
Male sex	0.559	0.257–1.215	0.142
BMI (per 1 kg/m^2^)	0.998	0.943–1.056	0.932
Diabetes mellitus	0.178	0.021–1.469	0.109
log(IL-1β) at admission	0.050	0.000–1617.9	0.571
log(IL-6) at admission	1.116	0.394–3.162	0.837
log(IL-17A) at admission	0.787	0.440–1.409	0.420
log(IL-12/IL-23) at admission	0.880	0.672–1.151	0.350
log(IL-28A) at admission	0.715	0.134–3.816	0.695

aOR, adjusted odds ratio; CI, confidence interval; BMI, body mass index; log, natural log-transformed; Reference, primary AF; None of the variables achieved *p* < 0.05. Model LLR *p* = 0.509.

## Data Availability

The data presented in this study are available from the corresponding author upon reasonable request due to the data protection restrictions of the clinic.

## Abbreviations

The following abbreviations are used in this manuscript:

AF	Atrial fibrillation
NNCS	Syzganov National Scientific Centre of Surgery
IL	Interleukin
RHiCA	Department of Interventional Cardiology, Arrhythmology and Endovascular Surgery
BMI	Body mass index
IQR	Interquartile range
